# Top Management Team Knowledge Hiding and Enterprise Innovation Performance: A Moderated Mediation Model

**DOI:** 10.3389/fpsyg.2021.783147

**Published:** 2021-12-20

**Authors:** Pengfei Rong, Shuang Liu

**Affiliations:** College of Philosophy, Law and Politics Science, Shanghai Normal University, Shanghai, China

**Keywords:** TMT, knowledge hiding, team creativity, team competitive climate, enterprise innovation performance

## Abstract

Top management team (TMT) knowledge hiding, which is not only related to the normal operation of the team but also closely related to enterprise innovation performance, has been paid little attention to in the previous studies. Based on the theories of upper echelons, knowledge management, and innovation, this study proposed a moderated mediation model to research how TMT knowledge hiding affected enterprise innovation performance. In this model, TMT knowledge hiding was the independent variable, TMT creativity was the mediating variable, enterprise innovation performance was the dependent variable, and team competitive climate was the moderating variable. MPLUS7.0 was used for the CFAs to evaluate the discriminate validities of the key variables, and SPSS 22.0 was used to calculate the descriptive statistics, analyze the correlations between variables, make the multiple regression analysis, and process the data obtained from 612 executives in 53 TMTs. The results showed that TMT knowledge hiding had a significant negative impact on enterprise innovation performance; TMT creativity had a partial mediating effect between TMT knowledge hiding and enterprise innovation performance, and team competitive climate had a moderating effect on the relationship between TMT knowledge hiding and TMT creativity. These research results fill up the gap of the theoretical research in TMT knowledge hiding and provide scientific guidance to reasonably reduce or eliminate the phenomenon of TMT knowledge hiding and improve enterprise innovation performance.

## Introduction

In the era of the knowledge economy, knowledge has become the most important production factor after going beyond the land and capital (Pan and Zhang, 2016). For an organization, its competitive advantage increasingly depends on effective knowledge management and organizational learning, and the economic benefits of the existing knowledge elements can be brought into full play only when knowledge gets promoted to the smooth flow within the organization (Mahdi et al., 2019). However, knowledge depends on the individuals in the organization, and the individuals’ knowledge activities are the key to the exertion of the knowledge utilities (Yi et al., 2021). For example, many enterprises have realized the importance of knowledge sharing and promoted it by strengthening the knowledge management system and using the information technology, but employes usually do not think the organization owns their “intellectual property rights” and the organization cannot force them to transfer their knowledge to other members. As a result, employes can freely choose to keep or hide their knowledge according to their actual needs, thus causing negative knowledge management activities within the organization, which is not conducive to the spread of knowledge (Singh et al., 2021). Therefore, how to stimulate the positive knowledge activities of the organization members and inhibit the negative knowledge activities, has increasingly become the focus of knowledge management theory and practice (Zhang and Sun, 2014). Unfortunately, the existing researches focus more on knowledge sharing (Ali et al., 2020; Singh et al., 2021), knowledge creation (Pao et al., 2020), and other positive knowledge activities (Wang et al., 2018) within certain organizations, while the researches on knowledge hiding (Chatterjee et al., 2021), knowledge misappropriation (Colombo and Piva, 2018) and other negative knowledge activities (Khalid et al., 2020) are still in its infancy. Among them, the phenomenon of knowledge hiding widely exists in various organizations (Bari et al., 2020) and often has adverse effects on organizational performance (Connelly et al., 2012), so it needs to be studied urgently.

Knowledge hiding is a kind of behavior that employes deliberately conceal or cover up knowledge in the face of their colleagues’ knowledge requests (Connelly et al., 2012). In the early stage, the scholars mainly focused on the phenomenon of knowledge hiding to individual employes and revealed that the antecedents of the knowledge hiding behavior were not only the leadership style of managers (Men et al., 2020) and the perceived knowledge ownership of employes (Pan and Zhang, 2016) but also the interpersonal distrust (Connelly et al., 2012) and workplace exclusion (Gao and He, 2019). Since then, organizational theory researchers had found that knowledge hiding existed in all types of organizations (Bari et al., 2020), which had a profound impact on the performance of the organizations (Arain et al., 2020). Therefore, they began to study the knowledge hiding behavior at the organizational level. Team knowledge hiding measures the knowledge hiding status of the whole team and reveals the vicious circle of knowledge hiding among team members (Li and Huang, 2018). Compared with the individual knowledge hiding behavior, team knowledge hiding is not only related to the psychological, cognitive, and other factors of team members, but also closely related to the interaction between team members, so it is more complex and difficult to accurately grasp. Therefore, the research on team knowledge hiding is still in its infancy, and no relevant research on the knowledge hiding behavior of the top management team (TMT) has been found.

Top management team is a small group of the senior managers, which is composed of CEO, general manager, deputy general manager, and senior managers who report to them directly, is responsible for making and implementing the decisions of innovation and development, and leads the sustainable and healthy growth of the enterprise (Rong and Ge, 2014). With the increasingly fierce competition, the business environments of the enterprises are becoming more and more complex, making TMT has to face many risks and uncertainty factors in the daily decision-making process. To mitigate the risks in decision-making, TMT must constantly acquire new knowledge from the outside and stimulate team creativity through knowledge sharing among TMT members, so as to realize the decision-making innovation and help the enterprise improve its innovation performance (Rong et al., 2019a). However, although some scholars have done the relevant researches on how to acquire knowledge (Zhang and Ge, 2016) and promote knowledge sharing within the TMT (Sperber and Linder, 2018) and proposed to build a knowledge management platform and standardize the knowledge sharing system to promote knowledge sharing, there are still some negative knowledge activities such as knowledge hiding, knowledge avoidance, and knowledge retention within the TMT, bringing about a profound impact on the decision-making process of TMT and enterprise innovation performance (Evan et al., 2015). Among the negative knowledge activities above, TMT knowledge hiding is the most common phenomenon (Bari et al., 2020). Therefore, this study is based on the existing researches of knowledge hiding and comprehensively uses the theories of upper echelons, knowledge management, and innovation to explore the problem of “how TMT knowledge hiding affects enterprise innovation performance,” with a view to making up for the deficiency of the existing studies which only focus on the positive knowledge activities but ignore the negative knowledge activities within TMT, assisting enterprises to strengthen TMT knowledge management and improving enterprise innovation performance.

## Theoretical Background

Both the theoretical analysis and the hypotheses of this study are based on the theories of upper echelons, knowledge management, and innovation. First of all, the research on TMT comes from the theory of upper echelons (Hambrick and Mason, 1984), so the analysis on the internal operation of TMT was made according to the theory; second, knowledge hiding belongs to the category of knowledge management (Vaio et al., 2021), so the analysis of TMT knowledge hiding used the theory of knowledge management; finally, given the effectiveness of the theory of innovation in analyzing enterprise innovation behaviors (Adro and Leitão, 2020), the analysis of enterprise innovation is based on this theory.

### Theory of Upper Echelons

The theory of upper echelons proposed by Hambrick and Mason (1984) holds that the characteristics of TMT will affect the organizational performance and strategic choice and that both the different cognitive bases, values, insights into TMT members, and the interaction process between these characteristics will affect the organizational competitive behavior. Therefore, the scholars have studied the genders (Quintana-García and Benavides-Velasco, 2016), ages (Bjornali et al., 2016), and tenures (Shin et al., 2016) of TMT members and their heterogeneities (Lui et al., 2019), but these researches can neither reveal the decision-making preferences of TMT members nor explain the decision-making behavior of TMT, and the researches on the relationship between TMT demographic characteristics and the organizational performance have not reached any relatively stable conclusion (Mehrabi et al., 2021), so Hambrick (1994) put forward the aggregation concept of behavior integration and focused the research on the internal operation process such as team conflict (Rong et al., 2019b) and team knowledge sharing (Wang et al., 2020) within TMT. This study belongs to the research category of TMT internal process and relies on the theory of upper echelons to explore the relationship between TMT knowledge hiding and enterprise innovation performance.

### Theory of Knowledge Management

Knowledge is the cognition, judgment, or skill acquired through learning, practice, or exploration, and knowledge management is the activities of planning and managing knowledge, the knowledge creation process, and the knowledge application (Vaio et al., 2021). According to the theory of knowledge management, knowledge management is to build a quantitative and qualitative knowledge system in an organization so that the information and knowledge in the organization can be continuously fed back to the knowledge system through the process of obtaining, creating, sharing, integrating, recording, accessing, updating, and innovating, for the purpose of shaping the cycle of the organizational wisdom formed by continuous accumulations of both the personal and organizational knowledge, which can be used in the enterprises and is helpful for the enterprises to make the correct decisions and adapt to the changes in the market (Adaileh et al., 2020). Knowledge management is a new management idea and method emerging in the era of the knowledge economy, and it is not only an important content of enterprise management but also an important skill for managers (Ode and Ayavoo, 2020). Therefore, based on the theory of knowledge management, this study explores the phenomenon of TMT knowledge hiding, extends the study of knowledge management to the field of TMT, and enriches the existing theory of knowledge management.

### Theory of Innovation

Under the guidance of the innovation strategy and driven by both market and technology, innovation is an activity to obtain the innovation achievements and market share and improve the success rate of innovation itself through the whole innovation process of the concept generation, product development, technology acquisition, and process innovation (Adro and Leitão, 2020). Andreeva et al. (2021) found that the innovation activities at the enterprise level are largely affected by the external environments and that innovation is the result of interaction and cooperation in many aspects. From the perspective of corporate governance, although TMT does not directly participate in the technological innovation activities of an enterprise, the process of making and implementing the strategic decisions for the enterprise contains the inherent creativities of the executives, and the creative thinking and innovation abilities of TMT members have a profound impact on enterprise strategic innovation and management innovation (Rong et al., 2019a). Based on the theory of innovation, this study reveals the relationships between TMT knowledge hiding, TMT creativity, and enterprise innovation performance and provides a new way to understand the change in enterprise innovation performance from the perspective of corporate governance.

## Theoretical Model and Hypotheses Development

**FIGURE 1:**
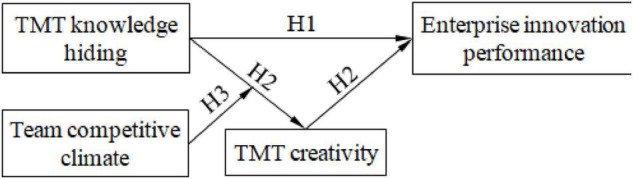
The theoretical model.

According to the changes in the internal and external environments, knowledge hiding is a flexible choice made by TMT members in the process of team operation (Arain et al., 2020). Once TMT members choose to hide their knowledge, this behavior will hinder the spread of more knowledge and information within the TMT and limit the creative thinking abilities of TMT members to put forward innovative decision-making schemes, which would affect the achievements of the innovation performance goals of the enterprise (Chatterjee et al., 2021). Specifically, when TMT members compete with each other and the team competitive climate is strong, they tend to hide their knowledge to maximize their personal or organizational interests, leading to a change in TMT creativity level (Wei and Ma, 2018). Therefore, this study proposes a moderated mediation model to research how TMT knowledge hiding affects enterprise innovation performance based on the theories of upper echelons, knowledge management, and innovation. In this model, TMT knowledge hiding is the independent variable, TMT creativity is the mediating variable, enterprise innovation performance is the dependent variable, and team competitive climate is the moderating variable. The model is shown in Figure 1.

According to the theoretical model, this study puts forward the following hypotheses to explore the internal influence mechanisms of TMT knowledge hiding on enterprise innovation performance.

### The Influence of Top Management Team Knowledge Hiding on Enterprise Innovation Performance

In an incentive market competition, to make the enterprise in an invincible position, TMT must take great efforts to carry out the management innovation or technological innovation activities to improve enterprise innovation performance (Rong et al., 2018). According to the theories of upper echelons and innovation, no matter what kinds of innovation activities, TMT needs to make the innovation decisions based on the brainstorming characterized by knowledge exchange and sharing to stimulate the enterprise’s innovation activities (Zhang and Zhu, 2021). However, the behavior of knowledge hiding hinders the transfer and communication of knowledge among TMT members, which makes TMT unable to share more knowledge. Phillips et al. (2017) found that team knowledge hiding will form the communication barriers and reduce team collaboration efficiency, making team members unable to effectively obtain and use the knowledge resources for research and innovation, thus reducing the quality of team decision-making and affecting the innovation behavior of the enterprise. The research of Faraj and Sproull (2000) also showed that team knowledge hiding not only directly hinders the rational use of knowledge, but also leads to team members’ inabilities to form a mental map which can help them to acquire the relevant knowledge, and both of them reduce team members’ abilities to quickly identify and solve the emerging problems. Although the behavior of knowledge hiding at the individual level can meet the individual needs of team members and help to improve their short-term performance (Connelly et al., 2012), in the long run, knowledge hiding at the team level will damage the performance of both organization and TMT, and it will lead to the decline of enterprise innovation performance (Chatterjee et al., 2021). Therefore, hypothesis 1 is proposed as follows:

H1: TMT knowledge hiding has a significant negative impact on enterprise innovation performance.

### The Mediating Effect of Top Management Team Creativity

Creativity is the ability to produce new ideas, discover and create new things, and it is the necessary psychological quality for people to successfully complete some creative activities (Emich and Vincent, 2020). In a complex and changeable business environment, the strategic decisions to be made and implemented by TMT often contain a large number of uncertain factors, which requires TMT to be creative and be able to creatively put forward some new ideas and methods to solve the new decision-making problems (Rong and Xie, 2021). TMT creativity is the ability for TMT members to present innovative and practical ideas for management practice and strategic decisions, and it is composed of TMT members’ knowledge, intelligence, and abilities (Shin and Zhou, 2007). According to the theory of knowledge management, to improve team creativity, TMT members need to constantly absorb and consolidate all kinds of knowledge, strive to expand their scopes of knowledge, and use knowledge to analyze and solve the various innovation decision-making problems (Nguyen et al., 2021), so as to promote the management innovation or technological innovation activities of the enterprise and improve enterprise innovation performance. Rong and Xie (2021) found that knowledge management within a TMT mainly depends on the interactive memory system, which is a division of labor system for TMT members to deal with the knowledge and information within the team. Though TMT interactive memory system plays an important role in improving the efficiency of knowledge management, enhancing team creativity, and the effectiveness of teamwork, it is vulnerable to the influence of TMT knowledge hiding. The previous studies have shown that the behavior of knowledge hiding at the team level is an obstacle to team interactive memory system, and it weakens the functions of team interactive memory system, reduces the level of knowledge management within the team (Li and Huang, 2018), and is not conducive to improving TMT creativity, which all reduce enterprise innovation performance (Chatterjee et al., 2021). Therefore, hypothesis 2 is proposed as follows:

H2: TMT creativity has a mediating effect on the relationship between TMT knowledge hiding and enterprise innovation performance.

### The Moderating Effect of Team Competitive Climate

According to the theory of knowledge management, employes have the right to decide whether they need to share their knowledge within the organization. However, the employes’ knowledge management activities are easily affected by the organizational climate and will alter with the change in the organizational climate (Connelly et al., 2012). Specifically in different team competitive climates, team members often choose how to manage their knowledge flexibly according to the degree of the internal competition within the team (Yang and Tang, 2018).

Team competitive climate refers to the sense of competition and pressure, which employes experience as a result of the team recognition and reward that the individuals get from the team based on the performance compared with other team members (Ye et al., 2020). Černe et al. (2014) found that team competitive climate will affect team members’ cognition, attitudes, and behaviors in the workplace, thus changing the behavior of team knowledge hiding. Because the “loss” of knowledge is likely to weaken the individual’s competitive advantage in the team (Yi et al., 2021), TMT members tend to show more mutual suspicions, mutual humiliations, and protections of the proprietary knowledge domains for their own interests in the fierce team competitive climate (NG, 2017), which destroys the basis of the mutual trusts among team members and makes them be more willing to choose to hide their knowledge by evasion and concealment, pretending to be stupid and make reasonable concealment when they face the knowledge requests from others. In addition, the research of Kilduff et al. (2016) found that the strong team competitive climate will stimulate the anxieties of TMT members about their internal positions in the team, team members who tend to compare themselves with their competitors and conduct more destructive and deceptive behaviors instead of helping others to maintain their competitive advantages. All of these promote the occurrence of knowledge hiding within TMT, make the knowledge and information shared in the team become limited, and restrain TMT creativity. However, when team competitive climate is weak, the senses of confrontation among TMT members are less, and the willingness of team cooperation is stronger. At the same time, TMT members trust each other, and both their knowledge domain awareness and knowledge hiding behavior also decrease (Wei and Ma, 2018). At this point, TMT members are more inclined to use cooperation instead of confrontation and competition, and they are more willing to share knowledge and information with each other and work together in the process of team decision-making so as to improve the level of team creativity. Therefore, hypothesis 3 is proposed as follows:

H3: Team competitive climate has a moderating effect on the relationship between TMT knowledge hiding and TMT creativity.

## Materials and Methods

To test whether the above hypotheses are tenable to judge the rationality of the theoretical model, this study follows the scientific research paradigm and makes an empirical analysis according to the following steps: first, the questionnaires were designed with reference to the existing maturity scales, and the data were collected by issuing and recovering the questionnaires; second, after the CFAs to evaluate the discriminate validity of the key variables using MPLUS 7.0, both the tests of common method deviation and data aggregation analysis were carried out to ensure the reliability and validity of the study; third, SPSS 22.0 was used for the descriptive statistical analysis, correlation analysis, and multiple regression analysis to complete the verification of the hypotheses, so as to judge the rationality of the theoretical model.

### Participants, Procedure, and Materials

In view of the universality of the research contents, this study did not select the TMTs in a specific industry to conduct the surveys but set the survey objects as TMT members of various types of enterprises. In addition, considering the high degree of enterprise innovation in the Yangtze River Delta in China, participants in this study were TMT members who were coming from all kinds of enterprises in the above areas. To ensure the recovery rate of the questionnaires, the surveys were conducted among the TMT members of different enterprises who were studying for an MBA or EMBA at the universities in Shanghai, China. Simultaneously, these MBA or EMBA participants also invited their senior colleagues who are the TMT members to fill out the questionnaires, and then, all the completed questionnaires were collected.

This study was approved by the Research Ethics Committee of Shanghai Normal University firstly, and all the participants provided written informed consents priors to taking part in the study. The anonymous questionnaires designed according to the existing mature scales were used to collect the data by means of probability sampling. To ensure the accuracy of the questionnaires, three senior experts in the field of human resource management were invited to check the contents of the survey items carefully. Then, the surveys were divided into two stages: the small-scale preinvestigations and the formal investigations. Among them, the small-scale preinvestigations conducted first were used to further examine the accuracy of the statements and the applicability of the survey items in the questionnaires. According to the method of the sampling surveys above, the small-scale preinvestigations with only six TMTs including 58 executives were conducted (since the small-scale preinvestigations were not used for the formal statistical analysis, the specific descriptions of these samples were omitted.) After checking the completed questionnaires carefully, the semantic expressions of four items in the questionnaires were modified, so as to make them easier for the participants to understand. Finally, to reduce the common methodological bias caused by the same participants or the consistent data sources, the orders of the measurement items were disrupted to reduce the self-defense consciousnesses of the participants, and the revised questionnaires were used to carry out the formal surveys. In the formal surveys, MBA or EMBA students who were TMT members of the enterprises were invited to fill in the questionnaires at first, and then they invited other TMT members of their enterprises to fill in the questionnaires. At last, all the questionnaires were collected and used for statistical analysis.

In this study, 54 TMTs participated in the formal surveys, 717 questionnaires were distributed, and 624 questionnaires were collected (87.03% response rate). After eliminating the invalid questionnaires such as similarity, blankness, and the aggregate value failing to meet the standard, 612 valid questionnaires from 53 TMTs were obtained (85.36% response rate). On average, each team consisted of 12 members (SD = 5.28, range 7–17). The descriptive statistics of samples for the formal surveys were shown in Table 1.

**TABLE 1:** The descriptive statistics of samples for the formal surveys.

Characteristic	Classification	Amount	Ratio
Gender	Male	346	56.54%
	Female	266	43.46%
Age	30–40 years old	103	16.83%
	41–50 years old	291	47.55%
	>50 years old	218	35.62%
Tenure	<3 years	74	12.09%
	3–5 years	173	28.27%
	6–10 years	213	34.80%
	>10 years	152	24.84%
Education level	Junior or below	72	11.76%
	Bachelor	329	53.76%
	Master or above	211	34.48%

### Measures

The main variables of the theoretical model included TMT knowledge hiding, TMT creativity, team competitive climate, and enterprise innovation performance, and the data used to measure these variables were obtained from questionnaires. To ensure that the reliability and validity could meet the requirements of the study, the questionnaires were designed for each variable to use a mature scale in the publicly available literature. Among them, the scale of TMT knowledge hiding developed by Connelly et al. (2012) was adopted, which included three dimensions, namely, evasion and concealment, pretending to be stupid, and the reasonable concealment, with a total of twelve items, a sample of which was “I promise to help later, but try to delay.” The scale of TMT creativity developed by Shin and Zhou (2007) was adopted, which included four items, a sample of which was “TMT members often produce the new suggestions and new ideas.” The scale of team competitive climate developed by Brown et al. (1998) was modified and adopted, which included four items, a sample of which was team leader will compare the performance of TMT members.” The scale of enterprise innovation performance developed by Wang (2011) was adopted, which included two dimensions, namely, management innovation and technology innovation, with a total of five items, a sample of which was “compared with the main competitors, the ability of the market management innovation for the enterprise is stronger.” All the scales were scored by Likert 5-point scale, from 1 to 5 represented “strongly disagree” to “strongly agree,” respectively, the greater the score was, the higher or stronger the situation was indicated by the measurement items. The operation results of each variable model and the fittings of the main indicators were shown in Table 2.

**TABLE 2:** The operation results of each variable model and the fittings of the main indicators.

Scale	Cronbach’s α	Square root	χ^2^/*df*	RMR	GFI	IFI	CFI	RMSEA
TMT knowledge hiding	0.86	0.77	2.18***	0.05	0.91	0.94	0.92	0.06
TMT creativity	0.84	0.82	2.09***	0.06	0.94	0.90	0.95	0.05
Team competitive climate	0.81	0.75	2.06***	0.07	0.92	0.95	0.92	0.06
Enterprise innovation performance	0.83	0.79	2.16***	0.06	0.90	0.94	0.92	0.06

***p < 0.001.

According to Table 2, it was found that the Cronbach’s α values of the scales were greater than 0.7, which indicated good internal consistency of the scales and high reliability; the square roots of the mean variances for the variables were greater than 0.70, which indicated the scales had the good convergence validity. In addition, the CFA analysis for the variables found that the normalized factor loads of the items were greater than 0.6 (*p* < 0.001), and the overall fits (χ^2^/*df*, RMR, GFI, IFI, CFI, and RMSEA) of the models for the variables were good, which indicated the items of the scales represented different constructs and had the discriminate validity. Generally, the scales of the variables had good reliability and validity and could meet the requirements of the study.

### Control Variables

The findings in a previous study had shown the genders, ages, tenures, and education levels of TMT members affected the operation process of TMT (Bengtsson et al., 2020). Accordingly, these demographic characteristics were taken as the control variables, and the relevant data were obtained for them in the survey items.

### Test of Common Method Deviation

This study used the single factor test of Harman to conduct exploratory factor analysis. After testing, it was found that the four factors were selected to explain 68.23% of total variances, and the first factor explained 21.76%. Thus, it could be seen there was no serious common methodological bias in this study.

### Data Aggregation Analysis

To aggregate the individual variable data of TMT members to the team level, the Rwg index was adopted to evaluate the consistency within the group of TMT knowledge hiding, TMT creativity, and team competitive climate, and the intraclass correlation coefficient (ICC) indexes (1) and (2) were used to estimate the heterogeneity between the groups. The results showed one of the team data could not be aggregated; after deleting them, the Rwg index medians of TMT knowledge hiding, TMT creativity, and team competitive climate in the remaining 53 teams were 0.92 (*M* = 0.90), 0.96 (*M* = 0.93), and 0.95 (*M* = 0.91), respectively, all of which showed the evaluations of the group members were consistent for each variable. The values of ICC (1) on TMT knowledge hiding, TMT creativity, and team competitive climate were 0.23, 0.21, and 0.24, and the values of ICC (2) were 0.64, 0.62, and 0.67. Therefore, the individual variable data of TMT knowledge hiding, TMT creativity, and team competitive climate could be aggregated to the team level.

## Results

Referring to the method of Zhao et al. (2020) and Ye et al. (2020), a multiple regression analysis is performed to assess the impact of TMT knowledge hiding on enterprise innovation performance, the mediating effect of TMT creativity on the relationship between TMT knowledge hiding and enterprise innovation performance, and the moderating effect of team competitive climate on the relationship between TMT knowledge hiding and TMT creativity. The data for the variables are centralized before the multiple regression analysis so as to further eliminate the multiple collinearities among the variables.

### Descriptive Analysis

Correlation analysis is a statistical analysis method to study the correlations between two or more random variables in the same status, and it is also the process to describe the closeness of the relationships between the variables and express it with the correlation coefficient (De Veaux et al., 2016). Table 3 shows the descriptive statistical results and the correlation coefficients between the main variables.

**TABLE 3:** The descriptive statistics and correlation coefficients for the variables (*N* = 53).

	1	2	3	4	5	6	7	8
(1) Gender								
(2) Age	0.06							
(3) Tenure	0.02	0.05						
(4) Education level	–0.03	–0.07	–0.10					
(5) TMT knowledge hiding	0.04	0.08	–0.02	0.07				
(6) TMT creativity	0.06	–0.03	−0.12*	0.11*	−0.44**			
(7) Team competitive climate	0.07	0.09	0.05	–0.04	0.36**	−0.22*		
(8) Enterprise innovation performance	0.08	−0.11*	–0.07	0.06	−0.49***	0.43***	−0.25*	
Mean	1.57	2.18	3.26	2.71	2.84	3.75	3.38	3.42
Standard deviation	0.72	0.69	1.06	0.80	0.83	1.12	0.94	0.88

*p < 0.05, **p < 0.01, ***p < 0.001.

According to Table 3 above, TMT knowledge hiding is significantly negatively correlated with TMT creativity (*r* = −0.44, *p* < 0.01) and enterprise innovation performance (*r* = −0.49, *p* < 0.001) and is significantly positively correlated with team competitive climate (*r* = 0.36, *p* < 0.01); TMT creativity is significantly negatively correlated with team competitive climate (*r* = −0.22, *p* < 0.05) and is significantly positively correlated with enterprise innovation performance (*r* = 0.43, *p* < 0.001); team competitive climate is significantly negatively correlated with enterprise innovation performance (*r* = −0.25, *p* < 0.05). Among the control variables, age is significantly negatively correlated with enterprise innovation performance (*r* = −0.11, *p* < 0.05); tenure is significantly negatively correlated with TMT creativity (*r* = −0.12, *p* < 0.05); education level is significantly positively correlated with TMT creativity (*r* = 0.11, *p* < 0.05). In general, the results of correlation analysis confirm the rationality of the series of hypotheses proposed in this study.

### Hypotheses Testing

Regression analysis is a statistical analysis method to determine the quantitative relationships among two or more variables, and it is a type of predictive modeling technology, which needs to analyze the specific forms of the correlations between the variables and determine their causal relationships (De Veaux et al., 2016). This study verifies the theoretical hypotheses through the multiple regression analysis and tests the mediating effect of TMT creativity between TMT knowledge hiding and enterprise innovation performance according to the causal step approach, and the results are shown in Table 4.

**TABLE 4:** The multiple regression analysis: the mediating effect of TMT creativity.

Variable	Dependent variable: enterprise innovation performance		
	Model 1	Model 2	Model 3
**Control variable**			
Gender	0.021	0.054	0.038
Age	−0.097	−0.074	−0.051
Tenure	−0.104	−0.095	−0.037
Education level	0.085	0.092	0.106
**Independent variable**			
TMT knowledge hiding		−0.437***	−0.305**
**Mediating variable**			
TMT creativity			0.329**
*R* ^2^	0.117*	0.271**	0.390**
*F*	2.762	27.615	36.141
Δ*R*^2^	0.104*	0.256**	0.367**
Δ*F*	4.688	34.922	42.355

*p < 0.05, **p < 0.01, ***p < 0.001.

According to the Model 2 Table 4, it can be seen TMT knowledge hiding has a significant negative effect on enterprise innovation performance (*r* = −0.437, *p* < 0.001), so hypothesis 1 is supported.

The moderating effect test of team competitive climate between TMT knowledge hiding and TMT creativity is shown in Table 5.

**TABLE 5:** The multiple regression analysis: the moderating effect of team competitive climate.

Variable	TMT creativity			
	Model 4	Model 5	Model 6	Model 7
**Control variable**				
Gender	0.026	0.052	0.047	0.056
Age	–0.044	–0.038	–0.033	–0.029
Tenure	–0.073	–0.062	–0.048	–0.037
Education level	0.065	0.070	0.075	0.066
**Independent variable**				
TMT knowledge hiding		−0.401***	−0.338**	−0.316**
**Moderating variable**				
Team competitive climate			−0.243**	−0.239**
**Product term**				
TMT knowledge hiding × Team competitive climate				−0.105*
*R* ^2^	0.047	0.139	0.276	0.319
*F*	2.719	17.165**	28.436**	34.604**
Δ*R^2^*	0.042	0.132	0.263	0.302
Δ*F*	2.661	21.256**	32.011**	40.928*

*p < 0.05, **p < 0.01, ***p < 0.001.

According to the Model 5 in Table 5, TMT knowledge hiding has a significant negative effect on TMT creativity (*r* = −0.401, *p* < 0.001); based on the Model 3 in Table 4, TMT creativity has a significant positive impact on enterprise innovation performance (*r* = 0.329, *p* < 0.01), and TMT knowledge hiding has a significant negative impact on enterprise innovation performance (*r* = −0.305), *p* < 0.01). Therefore, TMT creativity has a partial mediation between TMT knowledge hiding and enterprise innovation performance, and hypothesis 2 is supported.

**FIGURE 2:**
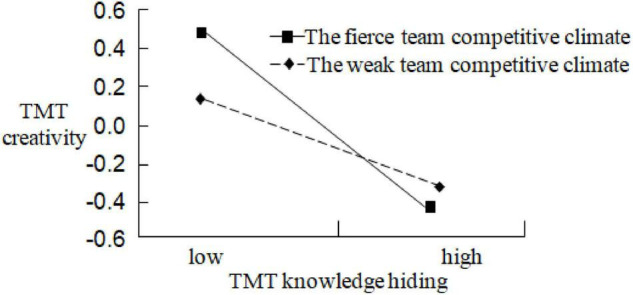
The moderating effect curves.

To test the moderating effect of team competitive climate between TMT knowledge hiding and TMT creativity, Model 7 introduces the product terms of TMT knowledge hiding with team competitive climate based on Model 6. The operation result of the Model 7 shows that the product terms of TMT knowledge hiding and team competitive climate are significantly related to TMT creativity (*r* = −0.105, *p* < 0.05), and it indicates that the moderating effect of team competitive climate between TMT knowledge hiding and TMT creativity is significant. Compared with Model 6, Model 7 improves significantly (Δ*F* = 40.928, *p* < 0.05), Δ*R*^2^ is 0.302, and therefore, team competitive climate has a negative moderating effect on the relationship between TMT knowledge hiding and TMT creativity. To further illustrate the moderating effect, this study establishes the coordinate axes based on a standard deviation above and below the mean, draws the moderating effect curves, and depicts the impact of TMT knowledge hiding on TMT creativity under the different levels of team competitive climate. The moderating effect curves are shown in Figure 2: in the case of the fierce team competitive climate, TMT knowledge hiding has a more negative impact on TMT creativity (*slope* = −0.773, *p* < 0.01); however, in the case of the weak team competitive climate, TMT knowledge hiding has a less negative impact on TMT creativity (*slope* = −0.364, *p* < 0.01). So, hypothesis 3 is supported.

## Conclusion and Discussion

### Conclusion

This study proposed a moderated mediation model to analyze the influence of TMT knowledge hiding on enterprise innovation performance, explore the mediating effect of TMT creativity between TMT knowledge hiding and enterprise innovation performance, and test the moderating effect of team competitive climate between TMT knowledge hiding and TMT creativity. Through the theoretical analysis and empirical research, the following conclusions were obtained: first, TMT knowledge hiding has a significant negative impact on enterprise innovation performance; second, TMT creativity has a partial mediating effect between TMT knowledge hiding and enterprise innovation performance; third, team competitive climate has a moderating effect on the relationship between TMT knowledge hiding and TMT creativity. Specifically, the fierce team competitive climate can strengthen the negative impact of TMT knowledge hiding on TMT creativity, whereas the weak team competitive climate can weaken the negative impact of TMT knowledge hiding on TMT creativity.

### Theoretical Contributions

Unlike the previous studies on employes’ knowledge hiding (Pan and Zhang, 2016; Men et al., 2020) or the general team knowledge hiding (Li and Huang, 2018; Arain et al., 2020), this study aims to explore the influence of TMT knowledge hiding on enterprise innovation performance and has made the new theoretical contributions as follows:

First, this study explored the negative impact of TMT knowledge hiding on enterprise innovation performance. The existing studies only focused on how TMT acquired and shared knowledge (Zhang and Ge, 2016; Sperber and Linder, 2018), but had not studied the negative knowledge activities within TMT. Based on the studies of Faraj and Sproull (2000) and Phillips et al. (2017), knowledge hiding was introduced into the field of TMT in this study to analyze how the negative knowledge management activities within TMT affected the changes in enterprise innovation performance. The results showed TMT knowledge hiding hindered the transfer and exchange of knowledge and information within TMT and had a significant negative impact on enterprise innovation performance. These findings further confirmed the researches of Fong et al. (2018) and Bari et al. (2020) and proved that the behavior of knowledge hiding was not conducive to enterprise innovation. It could be seen that this study broadened the research scope for the theory of knowledge management, revealed the important constraint of enterprise innovation performance from the perspective of corporate governance, and answered the question of how TMT knowledge hiding affected enterprise innovation performance.

Second, this study revealed the partial mediating effect of TMT creativity between TMT knowledge hiding and enterprise innovation performance. Team creativity is an important “port” for TMT to export (Zhao et al., 2020), and it is of great significance to study the role of TMT creativity in the relationship between TMT knowledge hiding and enterprise innovation performance, so as to reveal the specific paths of TMT knowledge hiding affecting enterprise innovation performance. This study showed TMT knowledge hiding not only directly acted on enterprise innovation performance, but also indirectly acted on it through TMT creativity. The findings further validated the views of Li and Huang (2018); Rong and Xie (2021) and indicated that the creative thinking abilities of TMT members had a significant impact on the process that TMT knowledge hiding affected enterprise innovation performance. It enriched the connotation for the theory of upper echelons and further explained the relationship between TMT knowledge hiding and enterprise innovation performance.

Third, this study found the moderating effect of team competitive climate between TMT knowledge hiding and TMT creativity. The previous studies have shown that context variables often play an important role in the internal operation of TMT, and the actual effects of TMT operation will be very different under the regulation of the different context variables (Chen and Liu, 2018; Aboramadan, 2021). Similar to the research of Černe et al. (2014), this study confirmed that team competitive climate could regulate the influence of TMT knowledge hiding on TMT creativity during the operation of TMT, the difference lied in the fact that team competitive climate was introduced into the specific organizational context of TMT knowledge hiding, and whether team competitive climate helped to adjust the impact of TMT knowledge hiding on TMT creativity. The research on the moderating effect of team competitive climate further broadened the application scope for the theory of knowledge management and provided a new situational perspective for revealing the process of knowledge management to TMT.

### Practical Contributions

The practical contributions of this study are mainly reflected in the following aspects:

First, the enterprise should attach great importance and strive to reduce the phenomenon of knowledge hiding within the TMT. Because the previous studies only focus on the positive knowledge management activities and have not studied the negative knowledge management behaviors within TMT, the enterprise lacks a comprehensive understanding of the process of knowledge management within TMT. The results of this study show that TMT knowledge hiding can negatively affect enterprise innovation performance. This conclusion reminds us that the enterprise should pay attention to both the positive knowledge management activities and the negative knowledge management activities such as knowledge hiding within TMT in the decision-making process and strive to reduce the negative impact of TMT knowledge hiding on enterprise innovation performance. As a result, the enterprise should pay much attention to the phenomenon of knowledge hiding within TMT, analyze the causes that make TMT members hide their knowledge, and take effective measures to avoid the behavior of knowledge hiding within TMT, so as to improve enterprise innovation performance in the complex and changeable business environments.

Second, this study reveals the important role of TMT creativity and points out the direction for the enterprise to enhance TMT creativity. The strength of TMT creativity is closely related to whether the enterprise can make the innovation decisions and achieve the innovation performance, so it is highly valued by the enterprise (Rong et al., 2019a; Zhao et al., 2020). This study not only finds that TMT creativity plays a part of the intermediary role in the process of TMT knowledge hiding affecting enterprise innovation performance, but also proves that the behavior of knowledge hiding is an important factor to inhibit TMT creativity in the decision-making process, and this points out the direction for the enterprise to improve TMT creativity. According to the conclusions of this study, the enterprise should try its best to reduce the phenomenon of TMT knowledge hiding in the decision-making process, encourage positive knowledge management activities within TMT, and promote the rapid exchange and sharing of all kinds of knowledge and information in the team, so as to enable TMT members to master the more abundant knowledge, fully stimulate their innovation vitalities, and constantly improve the overall creativity for TMT.

Finally, this study confirms the important moderating role of team competitive climate, which is conducive to guiding the enterprise to create more suitable working environments for the operation of TMT. In the previous studies, team competitive climate, the contextual variable, has rarely been concerned by scholars. However, this study finds the fierce team competitive climate can strengthen the negative impact of TMT knowledge hiding on enterprise innovation performance, leading to the increase of knowledge hiding behavior within TMT and the decline of enterprise innovation performance. Therefore, to reduce or eliminate the phenomenon of knowledge hiding within TMT, the enterprise should avoid creating a fierce team competitive climate. Instead of this, the enterprise should try its best to create a harmonious and friendly team working atmosphere, promote mutual understanding and trust among TMT members, and encourage team members to care for and cooperate with each other, so that TMT members can strive to form a team force, work together to do a good teamwork, and constantly improve the overall effectiveness of TMT.

### Limitations and Directions for the Future Research

The limitations of this study are mainly reflected in the following aspects. First, according to the previous studies, the phenomenon of knowledge hiding may occur at both the individual level and the organizational level. However, this study only explores the influence mechanisms of TMT knowledge hiding on enterprise innovation performance at the organizational level but does not study the behavior of knowledge hiding for TMT members at the individual level. Therefore, it is the direction for further research to construct a crosslevel model to analyze the effects of the knowledge hiding behavior coming from both top managers and TMT on organizational performance. Second, it is obvious that there are great differences between Chinese culture and Western culture, and the connotations of variables may be inconsistent under the different cultural backgrounds. However, this study follows the concepts and measurements of western scholars on TMT knowledge hiding, TMT creativity, and team competitive climate, but lacks the comparison of the connotations for these variables in the backgrounds of Chinese culture and Western culture. Therefore, both the comparative analysis of these variables above in different cultural backgrounds and measurement scales of TMT knowledge hiding, TMT creativity, and team competitive climate in the context of Chinese local culture are all urgently needed to be developed. Third, although this study collects the cross-sectional data through the questionnaires to verify the rationality of the theoretical model, the influence of TMT knowledge hiding on enterprise innovation performance and the moderating effect of team competitive climate are the dynamic processes, and their performance may vary at the different time points. Therefore, it is necessary to adopt the longitudinal research paradigm to further reveal the intrinsic process mechanisms dynamically.

## Data Availability Statement

The original contributions presented in the study are included in the article/supplementary material, further inquiries can be directed to the corresponding author.

## Ethics Statement

The studies involving human participants were reviewed and approved by the Research Ethics Committee of Shanghai Normal University. The patients/participants provided their written informed consent to participate in this study.

## Author Contributions

PR was responsible for the conceptualization of the idea and formulation of the overarching research goals, as well as the methodology, data curation, formal analysis, original draft, preparation, and funding acquisition. SL verified all results and created the figures, and also assisted with writing and editing of the manuscript. PR and SL reviewed and edited drafts of the manuscript. Both authors contributed to the article and approved the submitted version.

## Conflict of Interest

The authors declare that the research was conducted in the absence of any commercial or financial relationships that could be construed as a potential conflict of interest.

## Publisher’s Note

All claims expressed in this article are solely those of the authors and do not necessarily represent those of their affiliated organizations, or those of the publisher, the editors and the reviewers. Any product that may be evaluated in this article, or claim that may be made by its manufacturer, is not guaranteed or endorsed by the publisher.
